# Lipid profiles in patients with juvenile idiopathic arthritis: a systematic literature review and meta-analysis

**DOI:** 10.1186/s12944-023-01885-1

**Published:** 2023-08-25

**Authors:** Wen-Jia Zhao, Jiang-Hong Deng, Cai-Feng Li

**Affiliations:** Department of Rheumatology, Beijing Children’s Hospital, National Center for Children’s Health, Capital Medical University, Nan Li Shi Road No. 56, Beijing, 100045 China

**Keywords:** Juvenile idiopathic arthritis, Lipid profile, Meta-analysis, Rheumatology

## Abstract

**Objective:**

The purpose of this study was to comprehensively evaluate the lipid profiles in patients with juvenile idiopathic arthritis (JIA).

**Methods:**

The literature and relevant reviews were searched for published clinical studies on the relationship between JIA and blood lipid levels. The Newcastle–Ottawa scale (NOS) was applied to evaluate the risk and methodological value of the included case‒control and cohort studies. Standardized mean differences (SMDs) and 95% confidence intervals were derived for all variables with adequate unprocessed data. This meta-analysis followed the Preferred Reporting Items for Systematic Reviews and Meta-analysis (PRISMA) guidelines.

**Results:**

In total, 16 studies were incorporated through screening. The analysis findings revealed that the levels of very low-density lipoprotein cholesterol [SMD=-0.411, 95% CI (-0.774~-0.048), *P* = 0.026], high-density lipoprotein cholesterol [SMD=-0.528, 95% CI (-0.976~-0.079), *P* = 0.021], and apolipoprotein A1 [SMD=-1.050, 95% CI (-1.452~-0.647), *P* = 0.000] in JIA patients were statistically lower than those observed in healthy controls. The level of low-density lipoprotein cholesterol [SMD = 0.202, 95% CI (0.003 ~ 0.400), *P* = 0.046] was significantly higher in JIA patients than in healthy controls. In JIA patients, body mass index [SMD=-0.189, 95% CI (-0.690 ~ 0.311), *P* = 0.459], high-density lipoprotein [SMD =-1.235, 95% CI (-2.845 ~ 0.374), *P* = 0.133), low-density lipoprotein [SMD = 0.616, 95% CI (-0.813 ~ 2.046), *P* = 0.398), triglycerides (SMD = 0.278, 95% CI (-0.182 ~ 0.738), *P* = 0.236], total cholesterol [SMD=-0.073, 95% CI (-0.438 ~ 0.293), *P* = 0.696] and apolipoprotein B levels [SMD = 0.226, 95% CI (-0.133 ~ 0.585), *P* = 0.217] were not significantly different from those in healthy controls.

**Conclusions:**

The outcomes of this meta-analysis suggest that dyslipidemia is common in JIA patients compared to healthy controls. Patients with JIA have a significantly increased risk of atherosclerosis and cardiovascular disease later in life.

## Introduction

 Juvenile idiopathic arthritis (JIA) is a long-term rheumatic disorder that is mainly characterized by joint damage, which can be accompanied by multiple systemic systems [1]. The onset of the disease occurs before the age of 16 years, and the main feature of the disease is chronic synovitis [2]. Patients in advanced stages of the disease may experience complications such as impairment of the functions of the eyes, liver, spleen, and other organs and serious long-term health disorders, including physical disabilities [3]. The incidence of JIA ranges from 0.007 to 0.401%, affecting more females than males [4].

Much research evidence shows that long-term chronic inflammation affects lipid metabolism [5, 6]. The mechanism of dyslipidemia in JIA may be related to the chronic inflammatory process during the active phase of joint disease, the excessive release of some proinflammatory cytokines, and anti-rheumatic drug therapy [7]. Many studies [8–10] have demonstrated the presence of dyslipidemia in JIA patients during both acute and prolonged phases, yet findings across various investigations remain inconsistent. This study aims to measure and assess the existing research on lipid profiles in patients with JIA. The basic characteristics of dyslipidemia in JIA patients may preliminarily explore the mechanism of lipid metabolism disorder.

## Methods

This analysis was carried out in compliance with the Preferred Reporting Items for Systematic Reviews and Meta-analyses (PRISMA) regulations [11]. The protocol has been registered in PROSPERO (CRD42023445239).

### Inclusion and exclusion criteria

The criteria for inclusion were as follows: (1) cohort studies, case‒control studies, or cross-sectional studies evaluating the relationship between serum lipid levels and JIA. The diagnosis of JIA was made according to the consensus conference of the International League of Associations for Rheumatology (ILAR) in 2001 or the American College of Rheumatology (ACR) in 1986. The control group was composed of healthy children. (2) Quantitative measurements of blood lipids and the total number of cases were provided in the article. (3) Studies were published in either Chinese or English.

The exclusion criteria were as follows: (1) duplicate studies; (2) research papers or conference abstracts that could not be procured online; and (3) reviews and case series.

### Literature searches

Before designing the search strategy, the opinions of literature search experts were sought. From database establishment to July 15, 2023, under English and Chinese language restrictions, the MEDLINE, EMBASE, Cumulative Index to Nursing and Allied Health Literature, and Web of Science databases were systematically searched. The MEDLINE search strategy (Table 1) was modified to facilitate its application in other databases. Further relevant research was identified by manually searching the bibliographies of excluded review articles.

**Table 1:** Search syntax used for MEDLINE

Line	Syntax
1	(Juvenile adj2 arthritis).tw
2	Arthritis, Juvenile/
3	Lipid/
4	Fat/
5	Triglyceride/
6	Hyperlipemia/
7	Hyperlipoidemia/
8	Hypertriglyceridemia/
9	Hypercholesteremia/
10	Cholesterol/
11	Cholesterin/
12	Lipoprotein/
13	Apolipoprotein/
14	Lipid. tw
15	fat*.tw
16	cholest*.tw
17	weigh*.tw
18	1 or 2
19	3 or 4 or 5 or 6 or 7 or 8 or 9 or 10 or 11 or 12 or 13 or 14 or 15 or 16 or 17
20	18 and 19

### Data extraction

To ensure the quality of this meta-analysis, strict treatment control was carried out in the research design, literature retrieval and screening, data extraction, and data entry processes. The papers were retrieved, and then the content and data that met the criteria were sorted. The extraction of variables and inputting of data were independently conducted by two research members (J.-H.D., W.-J.Z.) back to back to ensure the quality of data entry. Conflicting literature or data that were difficult to determine were resolved by consultation or with the assistance of a third researcher (C.-F.L).

### Risk of bias (quality) assessment

The Newcastle–Ottawa scale (NOS) was applied to evaluate the risk and methodological value of the included case‒control and cohort studies [12]. The scale evaluates three research areas (selection criteria, comparability, and results) on a nine-point scoring system to indicate the level of quality achieved. To assess risk in the included cross-sectional surveys, the assessment standards were suggested by the Agency for Healthcare Research and Quality (AHRQ). Responses were evaluated as “Yes” (1 point), “No” (0 points), or “unclear” (0 points) [13]. A deviation risk table was created, and the research quality was summarized.

### Data synthesis and statistical analysis

The statistical software Stata (version 14.2) was utilized for synthesizing data and statistical interpretation [14]. To assist in the synthesis process, data were compressed through a dual approach. First, although all relevant data in the research reports were extracted, the comprehensive report only contains the most exhaustive evidence for the lipid profiles in each paper. Second, a single-point value was estimated for each variable in every study, and the confidence interval was 95%. The indicators in this meta-analysis were the mean and standard deviation of TC, TG, HDL and LDL levels, which were continuous variables. The detection methods in each study were different or did not provide the detection methods, so the standardized mean difference (SMD) was used for data analysis. If zero events were reported for one group in a comparison, a value of 0.5 was added to both groups for each study. All tests were 2-tailed, and P < 0.05 was considered statistically significant. Where possible, before meta-analysis, the ordered classification data were transformed into a single estimation through the implementation of the generalized trend least-squares method, as facilitated by the Stata glst module [15]. A random-effects model was used to pool the data, and statistical heterogeneity between summary data was evaluated utilizing Cochran’s Q-test and Higgin’s I^2^ value [16]. When *P* > 0.1 and I^2^ < 50%, there was no statistical heterogeneity among the studies, and the fixed effect model method was used to calculate the SMD value. Conversely, when *P* ≤ 0.1 or I^2^ ≥ 50%, the presence of statistical heterogeneity necessitates the employment of a random effect model for calculating the SMD value. Begg’s test and Egger’s test were used to test publication bias. Sensitivity analysis was performed by recombining and analyzing individual studies one by one.

## Results

### Study characteristics

The literature search obtained 1894 unique database records. Most of the studies (1739) were eliminated during the initial screening process based on their titles and summaries. Out of the 155 full-text studies that were scrutinized, only 16 studies [17–32] satisfied the predetermined inclusion criteria (Fig. 1; Table 2). Table 2 details the 2 case‒control studies, 7 cohort studies, and 7 cross-sectional studies incorporated in this meta-analysis. In total, this study encompassed 1502 participants, comprising 639 individuals diagnosed with JIA and 863 healthy controls. Most of the JIA patients included in the study were female, accounting for an average of 64.31%.

**Table 2:** Characteristics and design of the included studies

Study	Design	Country/ Region	Sample size		Age (mean ± SD)		Sex (female, %)		Disease duration (years)	Classification criteria	Diagnosis	Active disease (%)	Treatment at enrollment	Measurement
			Case	Control	Case	Control	Case	Control						
Hussain, 2020 [17]	CS	India	81	78	12.5(8–16)^a^	13(9–17)^a^	38.2	42	3 (2–6)^a^	ILAR criteria	JIA	42	Steroids, NSAIDs, and MTX	BMI VLDL LDL HDL
Jednacz, 2015 [18]	CS	Poland	30	20	14.0 ± 1.8	14.4 ± 1.8	76.7	75		ILAR criteria	JIA	67	Steroids, DMARDs, and TNF inhibitors	BMI TG TC HDL-C LDL-C
Breda, 2013 [19]	CC	Italy	38	40	7.05 ± 2.39	6.34 ± 2.25	68.4	55		ILAR criteria	JIA		NSAIDs and MTX	BMI TC TG HDL-C LDL-C
Pugliese, 2015 [20]	CS	Brazil	35	35	11.90 ± 2.0	12.50 ± 3.0			6.0 (0.25–13)^a^	ILAR criteria	JIA		Steroids, DMARDs, and TNF inhibitors	TC HDL LDL VLDL TG
Gonçalves, 2007 [21]	CS	Brazil	51	52	11.3 ± 4.2	12.5 ± 3.5	72.6	80.1		ILAR criteria	JIA		Steroids, NSAIDs, and DMARDs	HDL LDL VLDL TG
Tselepis, 1999 [22]	CO	France	26	22	11 ± 3	11 ± 4	53.9	63.6	4.5 (0.5–10)^a^	ACR criteria	JRA	53.9	Steroids, NSAIDs, and MTX	BMI HDL-C LDL-C TG TC Apo-A1 Apo-B
Bos, 2016 [23]	CC	Netherlands	76	131	10.4 ± 1.2	10.0 ± 1.4	66	63	3.6 ± 2.7	ILAR criteria	JIA		NSAIDs, and DMARDs	BMI
Więch, 2018 [24]	CS	Poland	46	46	12.74 ± 3.85	12.70 ± 3.80				ILAR criteria	JIA			BMI
Risum, 2019 [25]	CO	Norway	59	59	13.6 ± 2.2	13.5 ± 2.6	85	85	7.9 ± 3.8	ILAR criteria	JIA	33.9		BMI
Turoňová, 2018 [26]	CO	Slovakia	25	25	13.91 ± 2.98	14.01 ± 3.0	56	56	5.48 ± 3.91	ILAR criteria	JPSA		Steroids, NSAIDs, and DMARDs	HDL LDL TG TC
Studart, 2015 [27]	CO	Brazil	50	20	13.4 ± 4.4	11 ± 4	62	55	5.5 ± 4.3	ILAR criteria	JIA			BMI
Bakkaloglu, 1996 [28]	CS	Turkey	19	18						ACR criteria	JCA			BMI HDL LDL VLDL TG TC Apo-A1 Apo-B
Mani,2016 [29]	CO	USA	29	15	17.3 ± 6.7	21.7 ± 6.8	62.1	53.3		ILAR criteria	JIA		NSAIDs, and DMARDs	BMI HDL TG HDL-C LDL-C Apo-A1
Aranda-Valera,2020 [30]	CS	Spain	25	25	25.11 ± 7.21	27.21 ± 2.54	56	52	13.47 ± 5.47	ILAR criteria	JIA		Steroids, NSAIDs, DMARDs, and TNF inhibitors	BMI TG TC HDL-C LDL-C Apo-B
Sun,2019 [31]	CO	Mainland, China	22	45						ILAR criteria	sJIA	68.2		TG TC HDL-C LDL-C
Shen,2012 [32]	CO	Taiwan, China	27	232	14.58 ± 4.12	14.15 ± 3.76	40.7	37.5		ILAR criteria	JIA	46.6		BMI TG TC HDL-C LDL-C

*ACR *American College of Rheumatology, *Apo *Apolipoprotein, *BMI *Body mass index, *CC *a case‒control study, *CO *Cohort study, *CS *a cross-sectional study, *DMARDs *Disease-modifying anti-rheumatic drugs, *HDL *High-density lipoprotein, *HDL-C *High-density lipoprotein cholesterol, *ILAR *International League of Associations for Rheumatology, *JCA *Juvenile chronic arthritis, *JIA J*uvenile idiopathic arthritis, *JPSA *Juvenile psoriatic arthritis, *JRA *Juvenile rheumatoid arthritis, *LDL *Low-density lipoprotein, *LDL-C *Low-density lipoprotein cholesterol, *MTX *Methotrexate, *NSAIDs *Nonsteroidal anti-inflammatory drugs, *sJIA *Systemic juvenile idiopathic arthritis, *TC *Total cholesterol, *TNF*: tumor necrosis factor, *TG *Triglyceride, *VLDL *Very low-density lipoprotein

Note: ^a^Average age (upper quartile, lower quartile).

### Risk of bias

The incorporated papers exhibited varying degrees of quality, with scores ranging from five to eight. The scores were frequently downgraded due to the absence of independent verification of case status and insufficient follow-up measures (Tables 3, 4 and 5).

**Table 3:** Newcastle‒Ottawa Scale risk of bias assessment scores for case‒control studies

Study	Selection				Comparability		Exposure				Overall quality
	Adequate case definition with independent validation	Cases representative	Selection of community control participants	Controls have no history of disease outcome	Comparable on confounders		Determination of exposure factors		The same method of ascertaining cases and controls	Nonresponse rate same for both groups	
					study control for the most important factors	study control for the other important factors	Independent blind assessment	Record linkage			
Breda, 2013 [19]	1	1	0	1	1	0	0	1	1	0	6
Bos, 2016 [23]	1	1	1	1	1	0	0	1	1	0	7

**Table 4:** Newcastle‒Ottawa Scale risk of bias assessment scores for cohort studies

Study	Selection						Comparability		outcome					Overall quality
	Representativeness of exposed cohort		Selection of controls	Source of ascertainment		The outcome of interest was not present at the start of the study	Comparable on confounders		Outcome assessment		Adequate follow-up time (>5 years)	Adequacy of follow up		
	Truly	Somewhat		Reliable record	Structured survey		study control for the most important factors	study control for the other important factors	Independent blind assessment	Record linkage		Complete follow-up	>95% follow-up or description provided of loss to follow-up	
Tselepis, 1999 [22]	0	1	1	1	0	1	1	0	0	1	0	0	0	6
Risum, 2019 [25]	0	1	1	1	0	1	1	1	0	1	0	0	0	7
Turoňová, 2018 [26]	0	1	1	1	0	1	1	1	0	1	0	0	1	8
Studart, 2015 [27]	0	1	1	1	0	1	1	1	0	1	0	0	1	8
Mani, 2016 [29]	0	1	1	1	0	1	1	1	0	1	0	0	0	7
Sun, 2019 [31]	0	1	1	1	0	1	1	1	0	1	0	0	1	8
Shen, 2012 [32]	0	1	1	1	0	1	1	1	0	1	0	0	1	8

**Table 5:** Quality evaluation of cross-sectional studies by the Agency for Healthcare Research and Quality (AHRQ)

Study	1	2	3	4	5	6	7	8	9	10	11	Score
Hussain, 2020 [17]	Y	Y	N	Y	U	Y	N	Y	N	Y	N	6
Jednacz, 2015 [18]	Y	Y	Y	Y	U	Y	N	Y	N	Y	N	7
Pugliese, 2015 [20]	Y	Y	Y	Y	U	Y	N	Y	N	Y	N	7
Gonçalves, 2007 [21]	Y	Y	N	U	U	Y	N	Y	N	Y	N	5
Więch, 2018 [24]	Y	Y	N	Y	U	Y	N	Y	N	Y	N	6
Bakkaloglu, 1996 [28]	Y	Y	N	U	U	Y	N	Y	N	Y	N	5
Aranda-Valera, 2020 [30]	Y	Y	N	Y	U	Y	N	Y	N	Y	N	6

Note: (1) The data source was clear; (2) The inclusion and exclusion criteria of the exposed group and nonexposed group (case and control) were listed or referred to in previous publications; (3) The collection time of the research object was clear; (4) The research object was representative; (5) The measured variables were covered by other characteristics; (6) Describe any assessment to ensure quality; (7) The analysis results described the excluded objects; (8) Measures to evaluate and/or control confounding factors were described; (9) Describe the processing of lost data; (10) The response rate of patients and the integrity of data collection were summarized. 11. If there was a follow-up, the expected percentage of patients with incomplete data or follow-up results was written. Y: “Yes”; N: “No”; U: “Unclear”

### Body mass index (BMI)

A total of 11 studies [17–19, 22–25, 27, 29, 30, 32] surveyed the association between BMI and JIA. The heterogeneity result (*P* = 0.000, I^2^ = 92.9%) showed that there was a significant level of heterogeneity in the research literature, and the random effects model was employed for combined statistics. The results indicated a pooled SMD of -0.189, a 95% CI of -0.690 ~ 0.311, and a Z score of 0.74 (*P* = 0.459), suggesting no significant differences in BMI between JIA patients and healthy controls. To further analyze the sources of heterogeneity, sensitivity analysis was used to eliminate the studies one by one, and it was found that the analysis results were relatively robust. Subgroup analysis was conducted on potential regulatory variables. Subgroup analyses were performed according to the study design, and none of the results changed (Fig. 2; Table 6).

**Table 6:** Summary of meta-analysis results

Factors	Design	N	Heterogeneity test		Effect model	Outcomes		
			I^2^(%)	*P*		SMD(95%CI)	Z	*P*
BMI	CS	4	86.4	0.001	random	-0.736 (-1.987, 0.514)	1.15	0.248
	CC	2	0.0	0.659	random	-0.040 (-0.278, 0.199)	0.33	0.745
	CO	5	76.8	0.002	random	0.177 (-0.273, 0.627)	0.77	0.441
HDL	CS	4	88.8	< 0.001	random	-1.526 (-3.995, 0.944)	1.21	0.226
	CO	2	64.7	0.092	random	-0.652 (-1.379, 0.075)	1.76	0.079
LDL	CS	4	88.1	< 0.001	random	0.737 (-1.047, 2.520)	0.81	0.418
	CO	1	-	-	-	0.128 (-0.427, 0.683)	0.45	0.651
VLDL	CS	4	63.3	0.043	random	-0.411 (-0.774, -0.048)	2.22	0.026
TG	CS	5	80.5	0.001	random	-0.171 (-0.709, 0.368)	0.62	0.534
	CC	1	-	-	-	0.573 (0.119, 1.026)	2.48	0.013
	CO	5	91.0	< 0.001	random	0.687 (-0.164, 1.538)	1.58	0.114
TC	CS	4	62.1	0.048	random	0.060 (-0.407, 0.528)	0.25	0.800
	CC	1	-	-	-	0.522 (0.070, 0.973)	2.26	0.024
	CO	4	79.2	0.002	random	-0.362 (-0.949, 0.226)	1.21	0.228
HDL-C	CS	2	37.8	0.205	random	0.165 (-0.368, 0.697)	0.61	0.544
	CC	1	-	-	-	-0.154 (-0.598, 0.291)	0.68	0.499
	CO	4	64.7	0.037	random	-0.949 (-1.432, -0.466)	3.85	< 0.001
LDL-C	CS	2	0.0	0.476	fixed	0.236 (-0.182, 0.653)	1.11	0.269
	CC	1	-	-	-	0.651 (0.195, 1.107)	2.80	0.005
	CO	4	24.9	0.262	fixed	0.043 (-0.217, 0.303)	0.32	0.745
Apo-A1	CS	1	-	-	-	-1.438 (-2.165, -0.711)	3.87	0.001
	CO	2	69.3	0.071	fixed	-0.878 (-1.361, -0.395)	3.56	< 0.001
Apo-B	CS	2	46.9	0.170	fixed	0.180 (-0.243, 0.604)	0.83	0.404
	CO	1	-	-	-	0.341 (-0.334, 1.016)	0.99	0.322

*Apo *apolipoprotein, *BMI *Body mass index, *CC *A case‒control study, *CO *Cohort study, *CS *A cross-sectional study, *HDL *High-density lipoprotein, *HDL-C *High-density lipoprotein cholesterol, *LDL *Low-density lipoprotein, *LDL-C *Low-density lipoprotein cholesterol, *TC *Total cholesterol, *TG *Triglyceride, *VLDL *Very low-density lipoprotein

### High-density lipoprotein (HDL)

A total of 6 studies [17, 20, 21, 26, 28, 29] surveyed the association between HDL and JIA. The heterogeneity result (*P* = 0.000, I^2^ = 98.0%) showed that there was a significant level of heterogeneity in the research literature, and the random effect model was employed for combined statistics. The results indicated a pooled SMD of -1.235, a 95% CI of -2.845 ~ 0.374, and a Z score of 1.50 (*P* = 0.133), suggesting no significant differences in HDL cholesterol between JIA patients and healthy controls. To further analyze the sources of heterogeneity, sensitivity analysis was used to eliminate the studies one by one, and it was found that the analysis results were relatively robust. Subgroup analyses were performed according to the study design, and none of the results changed (Fig. 3; Table 6).

### Low-density lipoprotein (LDL) cholesterol

A total of 5 studies [17, 20, 21, 26, 28] surveyed the association between LDL cholesterol and JIA. The heterogeneity result (*P* = 0.000, I^2^ = 97.5%) shows that there was a significant level of heterogeneity in the research literature, and the random effect model was utilized for combined analysis. The results indicated a pooled SMD of 0.616, a 95% CI of -0.813 ~ 2.046, and a Z score of 0.84 (*P* = 0.398), suggesting no significant differences in LDL cholesterol levels between JIA patients and healthy controls. To further analyze the sources of heterogeneity, sensitivity analysis was used to eliminate the studies one by one, and it was found that the analysis results were relatively robust. Subgroup analyses were performed according to the study design, and none of the results changed (Fig. 4; Table 6).

### Very low-density lipoprotein (VLDL) cholesterol

A total of 4 studies [17, 20, 21, 28] surveyed the association between VLDL cholesterol and JIA. The heterogeneity result (*P* = 0.043, I^2^ = 63.3%) showed that there was some homogeneity in the research literature, and the random effect model was implemented for combined statistics. The results indicated a pooled SMD of -0.411, a 95% CI of -0.774~-0.048, and a Z score of 2.22 (*P* = 0.026), suggesting that the VLDL cholesterol level of JIA patients was statistically less than that of healthy controls (Fig. 5; Table 6). To further analyze the sources of heterogeneity, sensitivity analysis was used to eliminate the studies one by one, and it was found that the analysis results were relatively robust. All four study designs were of the same type, so no subgroup analysis was conducted.

### Triglyceride (TG)

A total of 11 studies [18–22, 26, 28–32] surveyed the association between TGs and JIA. The heterogeneity result (*P* = 0.000, I^2^ = 87.8%) showed that there was some homogeneity in the research literature, and the random effect model was employed for combined statistics. The results indicated a pooled SMD of 0.278, a 95% CI of -0.182 ~ 0.738, and a Z score of 1.18 (*P* = 0.236), suggesting no significant differences in TG levels between JIA patients and healthy controls. To further explore the potential sources of heterogeneity, subgroup analysis was conducted on potential regulatory variables. Subgroup analyses were performed according to the study design. Subgroup analyses were performed according to the study design. The results found significant differences in the case‒control study and no differences in the other types (Fig. 6; Table 6).

### Total cholesterol (TC)

A total of 9 studies [17, 19, 20, 22, 26, 28, 30–32] surveyed the association between TC and JIA. The heterogeneity result (*P* = 0.000, I^2^ = 76.1%) showed that there was some homogeneity in the research literature, and the random effect model was utilized for combined statistics. The results indicated a pooled SMD of -0.073, a 95% CI of -0.438 ~ 0.293, and a Z score of 0.39 (*P* = 0.696), suggesting no significant differences in TC levels between JIA patients and healthy controls. To further explore the potential sources of heterogeneity, subgroup analysis was conducted on potential regulatory variables. Subgroup analyses were performed according to the study design. The results found significant differences in the case‒control study and no differences in the other types (Fig. 7; Table 6).

### High-density lipoprotein cholesterol (HDL-C)

A total of 7 studies [18, 19, 22, 29–32] surveyed the association between HDL-C and JIA. The heterogeneity result (*P* = 0.000, I^2^ = 78.1%) showed that there was some homogeneity in the research literature, and the random effect model was implemented for combined statistics. The results indicated a pooled SMD of -0.528, a 95% CI of -0.976~-0.079, and a Z score of 2.31 (*P* = 0.021), suggesting that the HDL-C level of JIA patients was statistically lower than that of healthy controls. To further explore the potential sources of heterogeneity, subgroup analysis was conducted on potential regulatory variables. Subgroup analyses were performed according to the study design. The results found significant differences in the cohort studies and no differences in the other types (Fig. 8; Table 6).

### LDL-C

A total of 7 studies [18, 19, 22, 29–32] surveyed the association between LDL-C and JIA. The heterogeneity result (*P* = 0.138, I^2^ = 38.1%) showed that there was little homogeneity in the research literature, and the fixed effect model was utilized for combined statistics. The results indicated a pooled SMD of 0.202, a 95% CI of 0.003 ~ 0.400, and a Z score of 1.99 (*P* = 0.046), suggesting that the LDL-C level of JIA patients was statistically higher than that of healthy controls. To further explore the potential sources of heterogeneity, subgroup analysis was conducted on potential regulatory variables. Subgroup analyses were performed according to the study design. The results found significant differences in the case‒control study and no differences in the other types (Fig. 9; Table 6).

### Apolipoprotein (Apo)-A1

A total of 3 studies [22, 28, 29] surveyed the association between Apo-A1 and JIA. The heterogeneity result (*P* = 0.950, I^2^ = 0.0%) showed that there was little homogeneity in the research literature, and the fixed effect model was employed for combined statistics. The results indicated a pooled SMD of -1.050, a 95% CI of -1.452~-0.647, and a Z score of 5.11 (*P* = 0.000), suggesting that the Apo-A1 level of JIA patients was statistically inferior to that of healthy controls. To further explore the potential sources of heterogeneity, subgroup analysis was conducted on potential regulatory variables. Subgroup analyses were performed according to the study design, and none of the results changed (Fig. 10; Table 6).

### Apo-B

A total of 3 studies [22, 28, 30] surveyed the association between Apo-B and JIA. The heterogeneity result (*P* = 0.361, I^2^ = 1.9%) showed that there was little homogeneity in the research literature, and the fixed effect model was employed for combined statistics. The results indicated a pooled SMD of 0.226, a 95% CI of -0.133 ~ 0.585, and a Z score of 1.23 (*P* = 0.217), suggesting no significant differences in Apo-B between JIA patients and healthy controls. Subgroup analyses were performed according to the study design, and none of the results changed (Fig. 11; Table 6).

### Sensitivity analysis and publication bias test

Sensitivity analysis did not identify any studies that caused heterogeneity in this study, and the results of this study are stable and reliable (Fig. 12). Begg’s and Egger’s bias tests and funnel plots showed no obvious asymmetry, indicating that there was little publication bias in the selected studies (Figs. 13 and 14).

## Discussion

The differences in serum lipid levels between the JIA and control groups were compared in this meta-analysis. In this meta-analysis of 16 trials involving 1502 participants, the results showed that the VLDL, HDL-C, and Apo-A1 levels in the JIA group were lower than those observed in the control group, and the LDL-C level in the JIA group was higher than that in the control group. There were no significant differences in BMI, HDL, LDL, TG, TC, and Apo-B between the JIA group and the control group.

In recent years, many studies have reported that compared with healthy children, children with JIA have blood fat metabolism disorders [33]. Current studies suggest that the inflammatory state and overexpression of proinflammatory cytokines caused by immune disorders in children with JIA at the active stage may lead to lipid metabolism disorders [34]. Chronic systemic inflammation in JIA patients can also induce changes in the structure and level of lipoproteins. Studies on dyslipidemia in children with JIA have shown different results. IIowite et al. [35] first described the changes in blood lipids in children with JIA, characterized by decreased HDL-C levels and increased TG and VLDL levels. In contrast, Gonvalves et al. [21], in a study of 51 children with JIA, found lower HDL-C levels and lower TG and VLDL levels in children with JIA than in healthy controls. Bohr et al. [36] reported that children with JIA had no obvious dyslipidemia, and TC, LDL-C, and HDL-C levels were within the normal range. However, Marangoni et al. [37] showed that plasma triglyceride, TC, and LDL-C levels were elevated and HDL-C levels were decreased in children with multiarticular JIA. This difference may be related to the JIA subtype and disease activity and to JIA treatment drugs. Glucocorticoids can increase the levels of all lipid components, while TNF-α antagonists and methotrexate may improve lipid metabolism [38].

VLDL is one of the plasma lipoproteins. VLDL may be related to physical activity and disease activity in JIA [39]. Some studies have shown that Apo-A1 is negatively related to JIA disease activity [40]. Apo-A1 can inhibit the activation of monocytes and macrophages and release interleukin-1, TNF-α, and other inflammatory factors [41]. In the process of inflammation in JIA, the level of SAA is significantly increased and replaces Apo-A1, resulting in a decrease in Apo-A1 [42].

## Study strengths and limitations

The literature included in this study was all case‒control studies or cohort studies published in the last 30 years, and the NOS evaluation criteria were applied to score each study. The criteria for the diagnosis and exposure of JIA were clear in the included literature, so the bias caused by disease diagnosis errors and the determination and measurement errors of exposure factors was low. The funnel plot also suggested low publication bias.

This study, nonetheless, is not without its constraints. First, bibliographic bias occurs in the process of literature collection. The language scope of the included studies was restricted to Chinese and English; thus, valuable research published in various linguistic versions could not be retrieved or evaluated. Second, all the papers included have been previously published, and unpublished research results and documentary evidence from other nontraditional sources were lacking. The literature with “positive” results may overstate the strength of the association for some risk factors. An analysis of the causes showed that publication bias may be related to the failure to retrieve unpublished literature with negative results, database bias, etc. Due to publication bias, the results may exaggerate the association strength of a risk factor and deviate from the authenticity of JIA. Therefore, this conclusion is clinically relevant for reference. Finally, the included case‒control and cross-sectional studies were observational, and the strength of evidence was low. The quality assessment of the incorporated studies based on NOS and AHRQ evaluation criteria showed that the character of each study was different, and the assessment of some literature was low.

## Conclusions

This review highlights studies of serum lipids in JIA. The outcomes of this meta-analysis suggest that dyslipidemia is common in JIA patients compared to healthy controls. The inflammatory status in JIA patients during the active phase of the disease or previous medication use may lead to lipid metabolism disorders. Patients with JIA have a significantly increased risk of atherosclerosis and cardiovascular disease later in life. Therefore, in clinical work, early intervention in lowering blood lipids can be performed on JIA patients with hyperlipidemia, thereby improving the prognosis and quality of life of patients. In future studies, it is still necessary to further analyze whether there is an interaction between different lipid indexes and to conduct a larger sample size population cohort study to explore the pathophysiological mechanism of lipid disorders in JIA.

## Data Availability

The authors confirm that the data supporting the findings of this study are available within the article.
