# Correction: Lipid profiles in patients with juvenile idiopathic arthritis: a systematic literature review and meta‑analysis

**DOI:** 10.1186/s12944-023-01913-0

**Published:** 2023-09-08

**Authors:** Wen‑Jia Zhao, Jiang‑Hong Deng, Cai‑Feng Li

**Affiliations:** Department of Rheumatology, Beijing Children’s Hospital, National Center for Children’s Health, Capital Medical University, Nan Li Shi Road No. 56, Beijing, 100045 China

**Correction: Lipids in Health and Disease 22, 136 (2023)**

**https://doi.org/10.1186/s12944-023-01885-1**

Following publication of the original article [1], the authors noticed that all figures 1, 2, 3, 4, 5, 6, 7, 8, 9, 10, 11, 12, 13 and 14 were missing due to typesetting mistake. The figures are as follows:

**Fig. 1:**
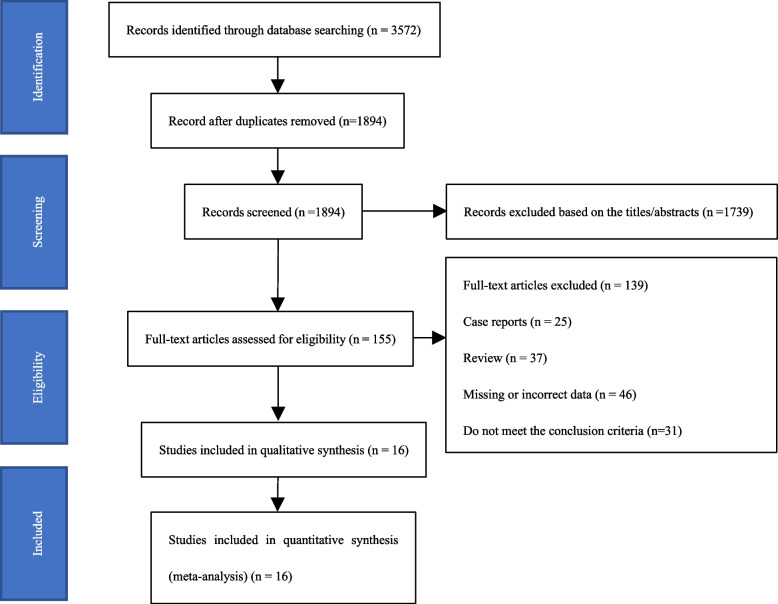
Processing of the studies extracted for meta-analysis

**Fig. 2:**
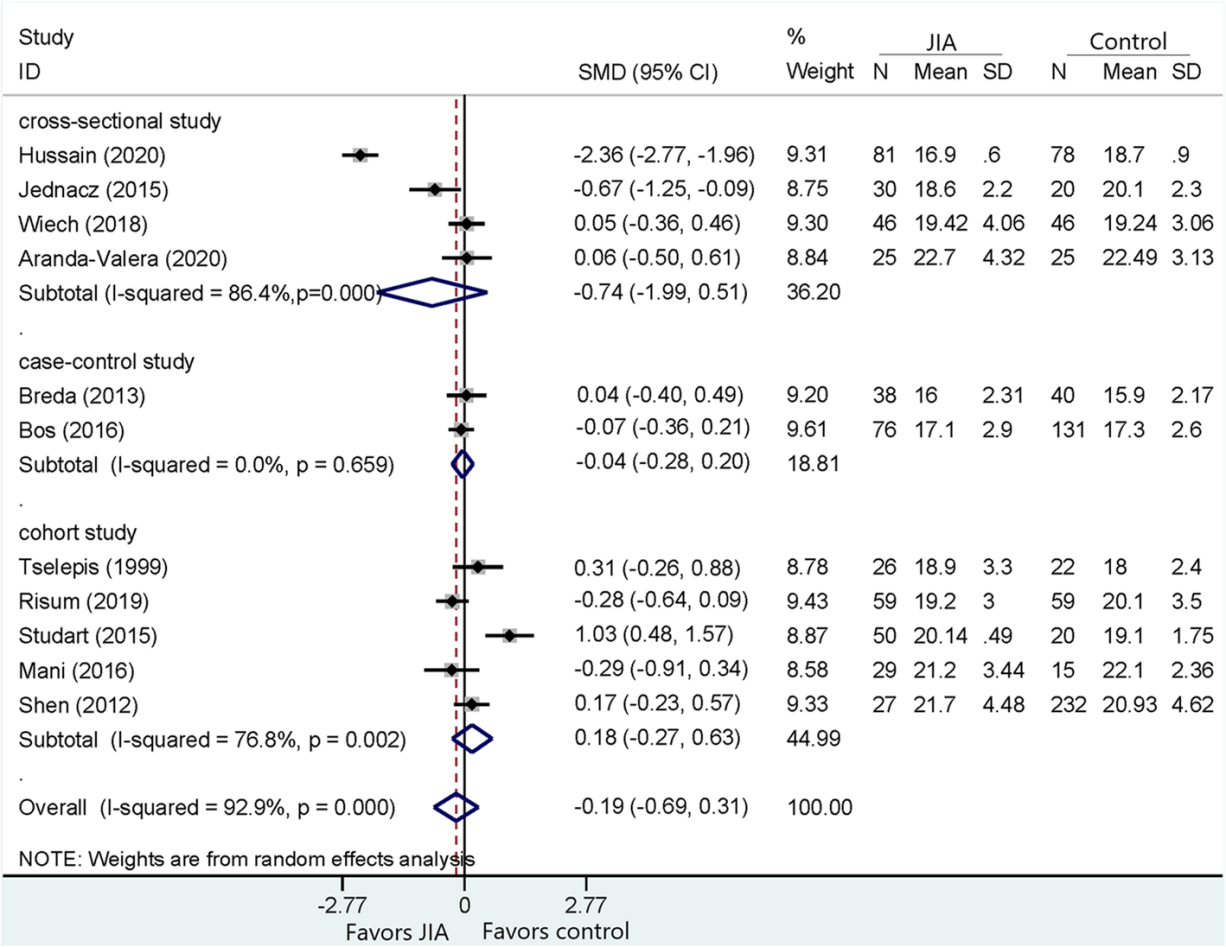
Comparison of MBI between JIA and healthy controls

**Fig. 3:**
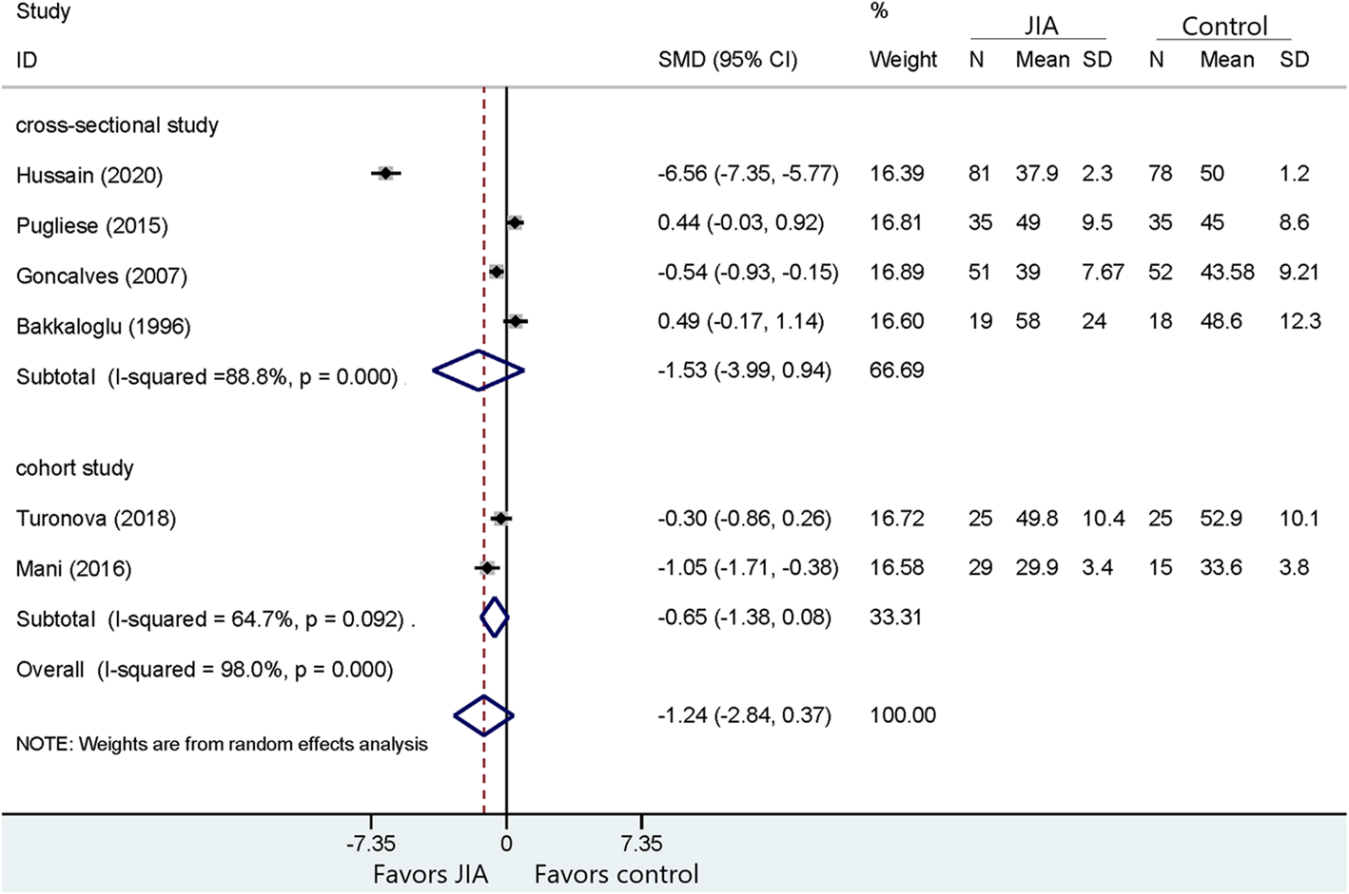
Comparison of HDL between JIA patients and healthy controls

**Fig. 4:**
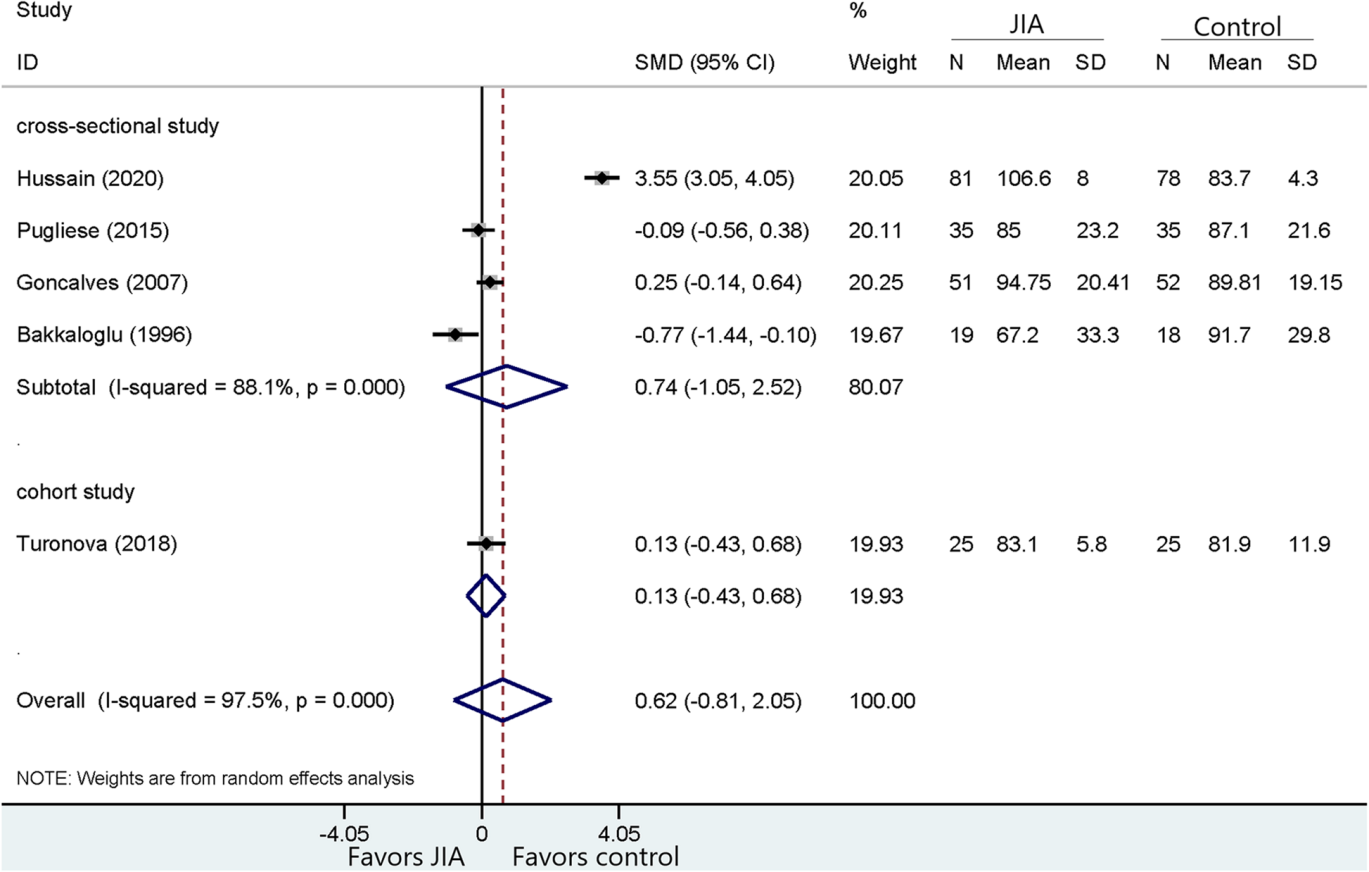
Comparison of LDL between JIA patients and healthy controls

**Fig. 5:**
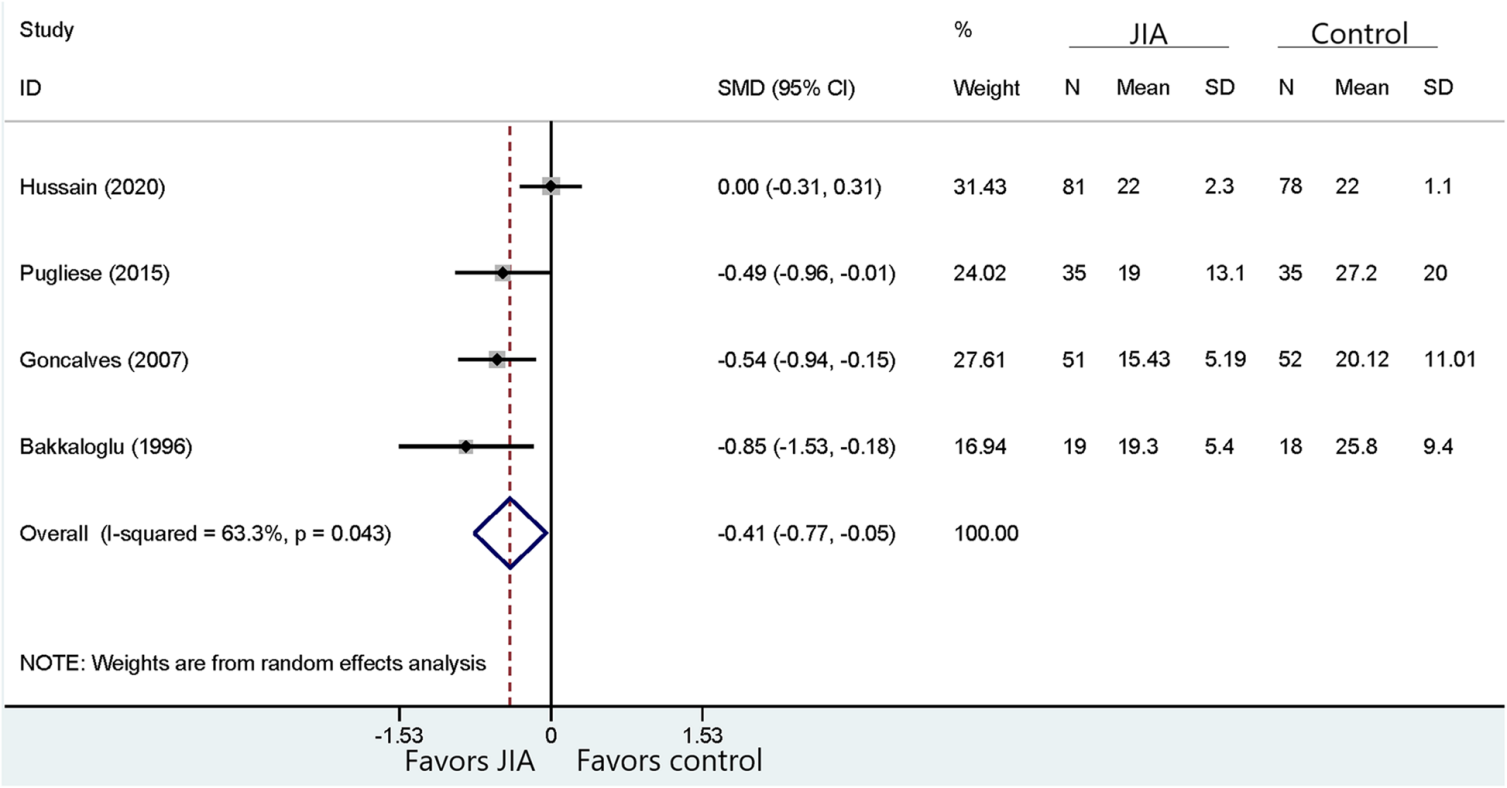
Comparison of VLDL between JIA and healthy controls

**Fig. 6:**
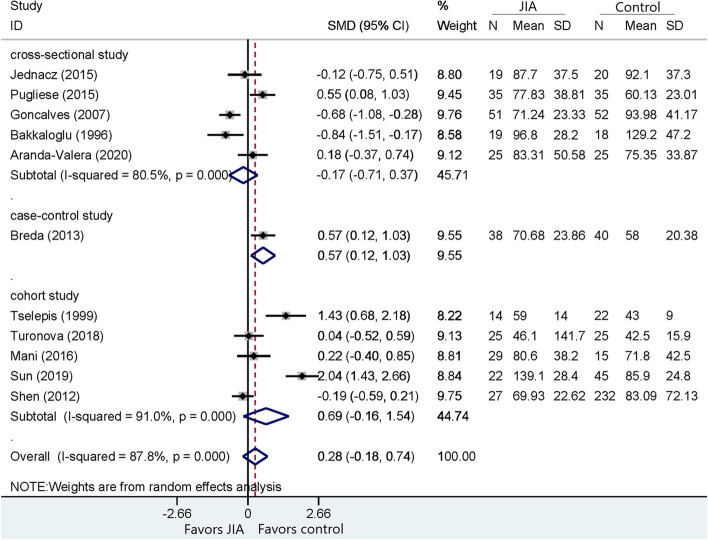
Comparison of TG between JIA and healthy controls

**Fig. 7:**
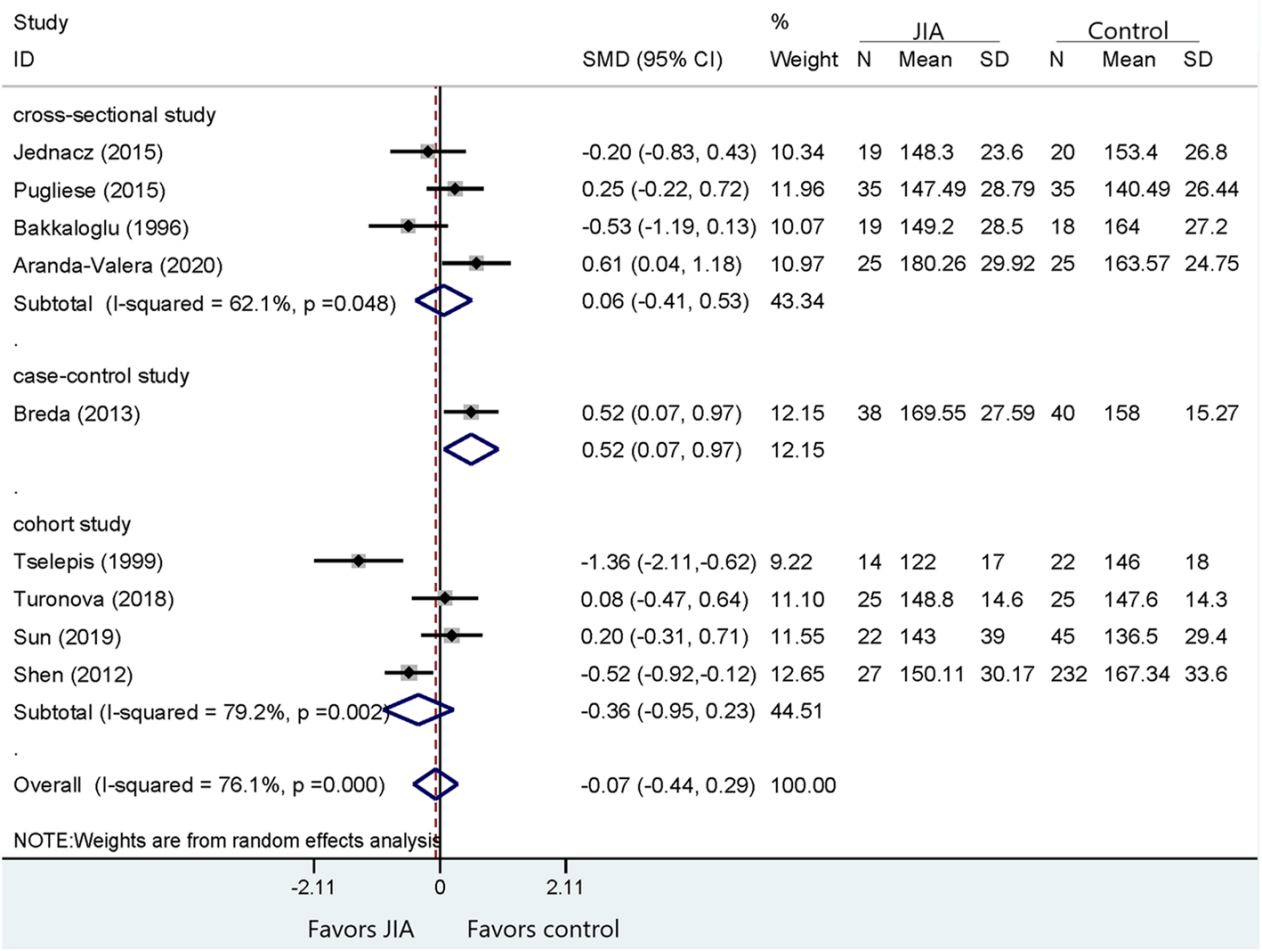
Comparison of TC between JIA patients and healthy controls

**Fig. 8:**
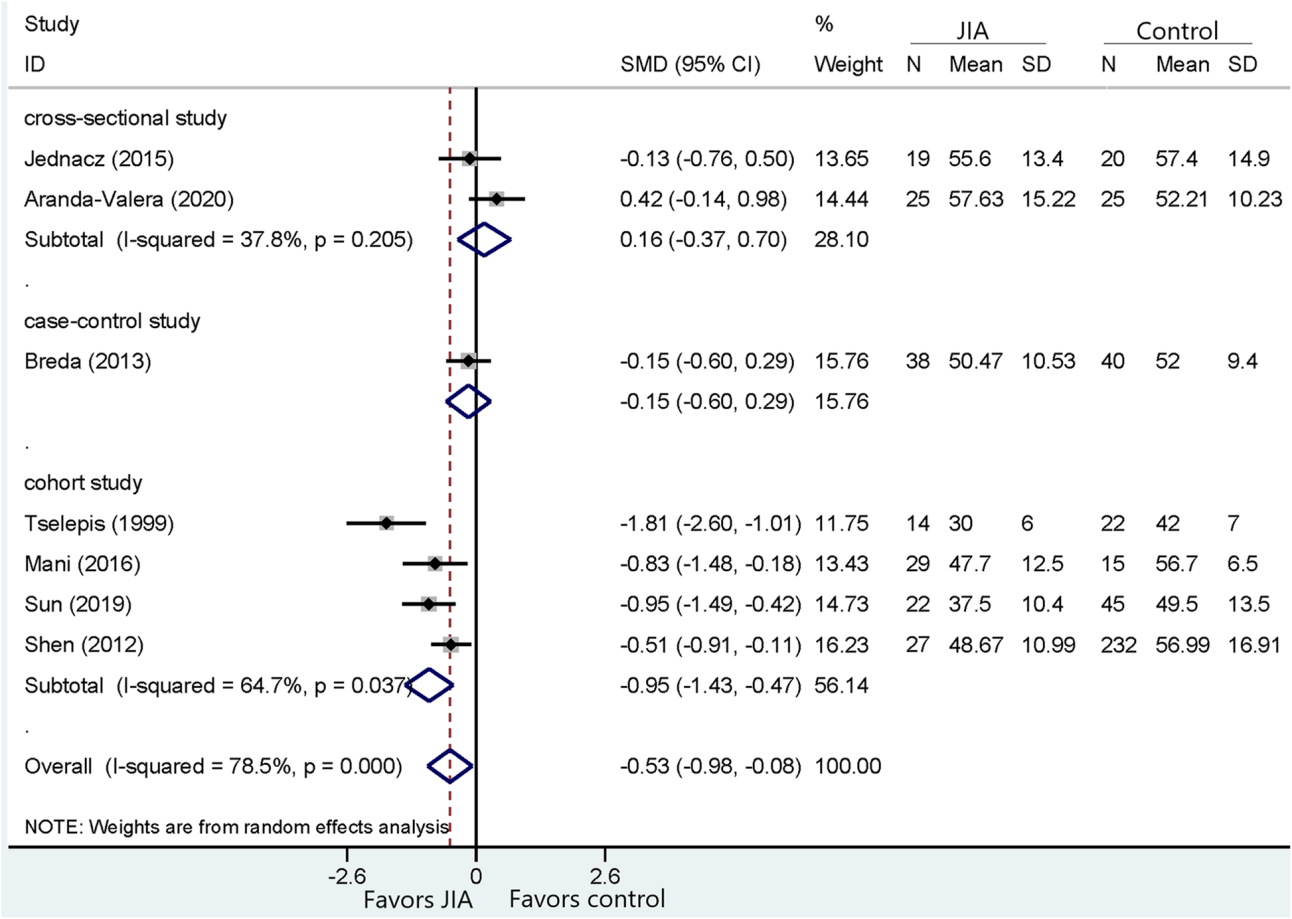
Comparison of HDL-C between JIA and healthy controls

**Fig. 9:**
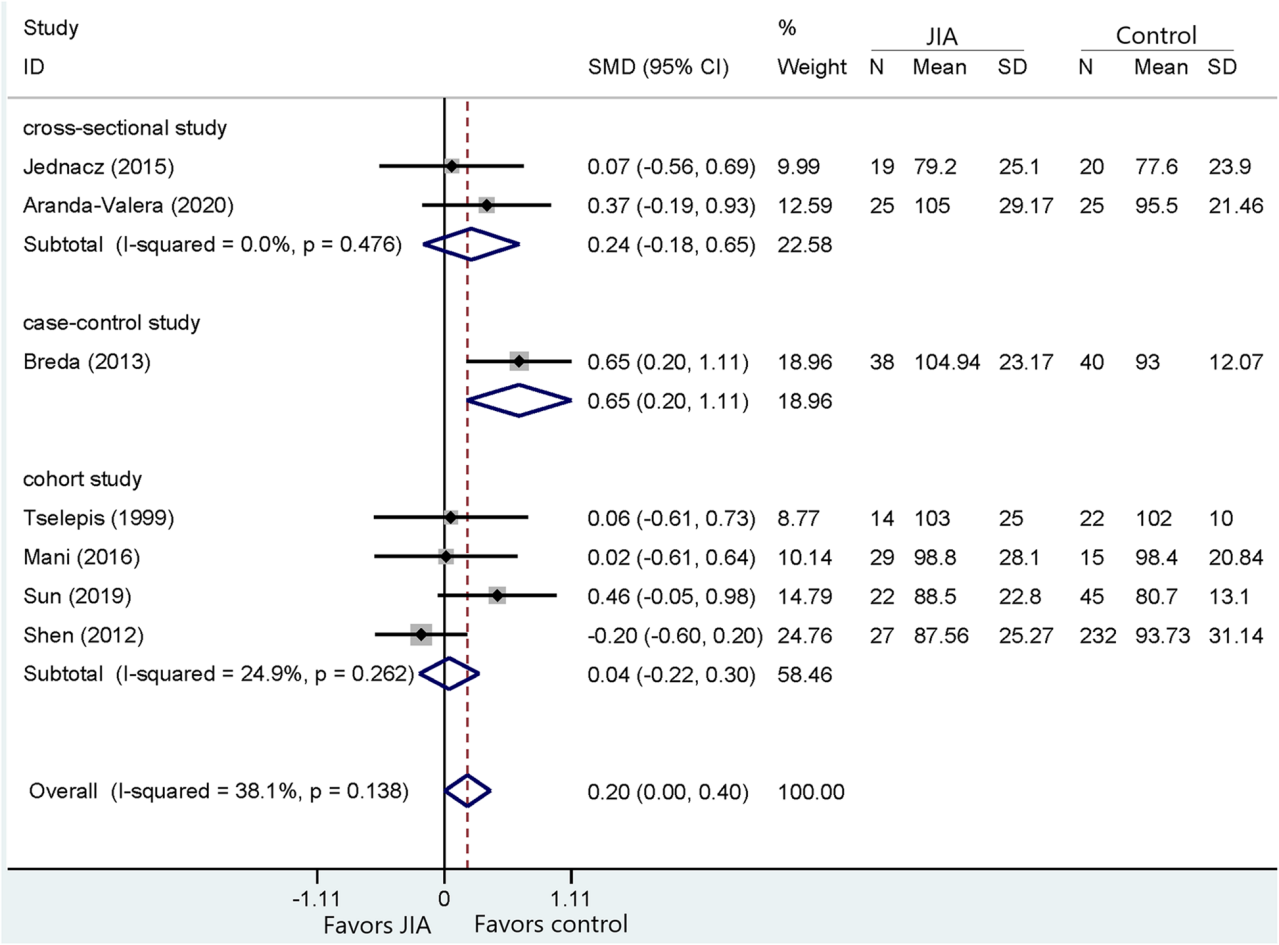
Comparison of LDL-C between JIA patients and healthy controls

**Fig. 10:**
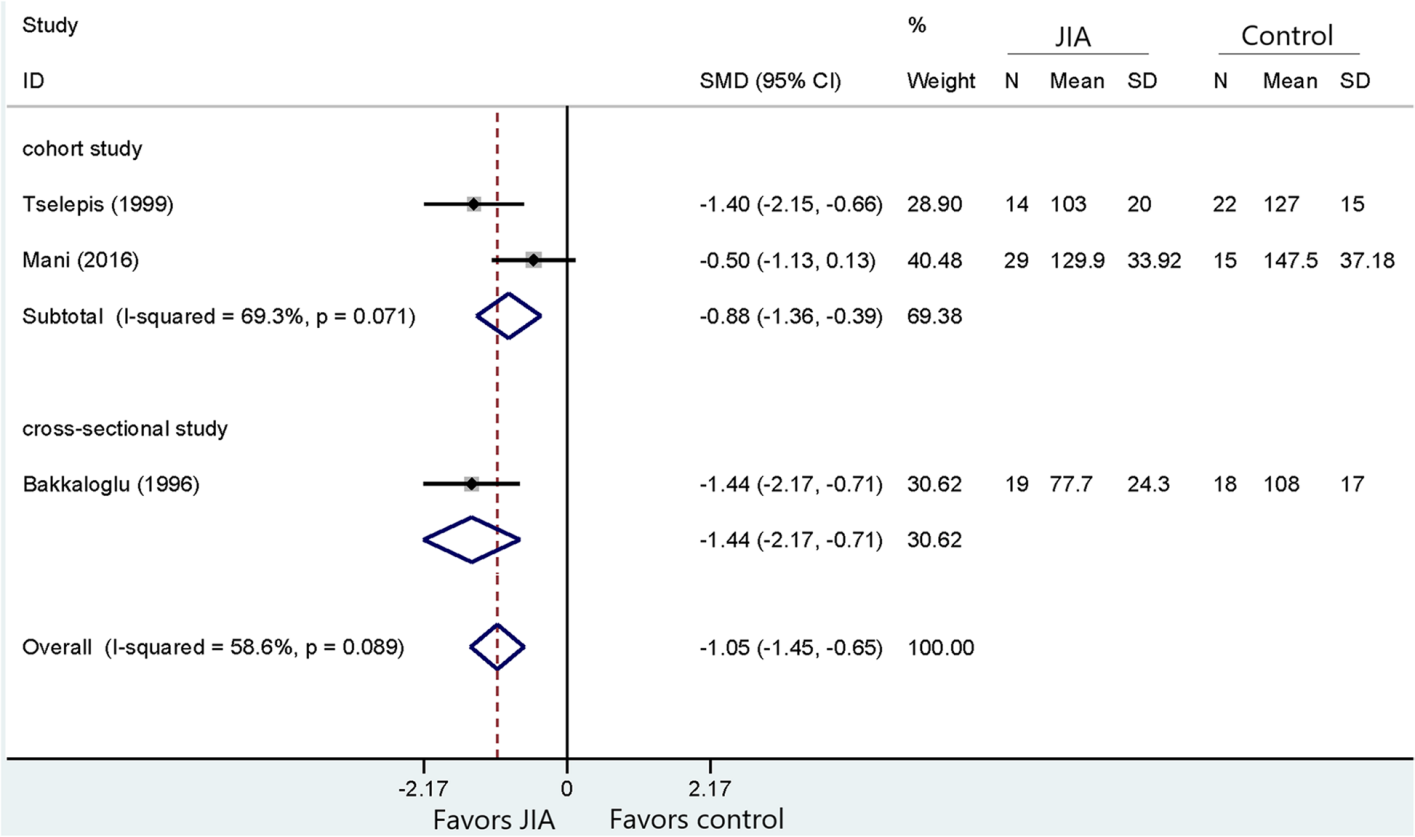
Comparison of Apo-A1 between JIA and healthy controls

**Fig. 11:**
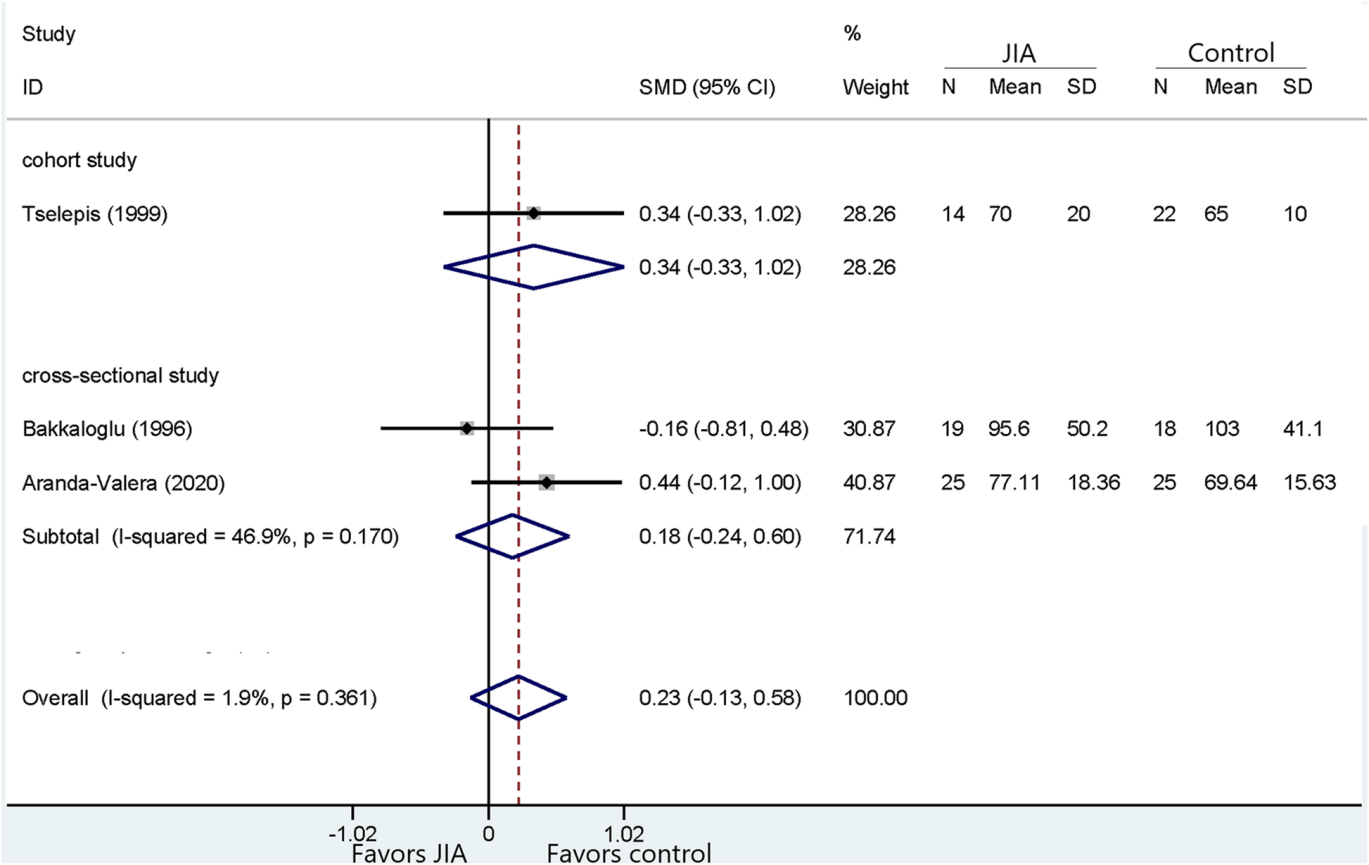
Comparison of Apo-B between JIA and healthy controls

**Fig. 12:**
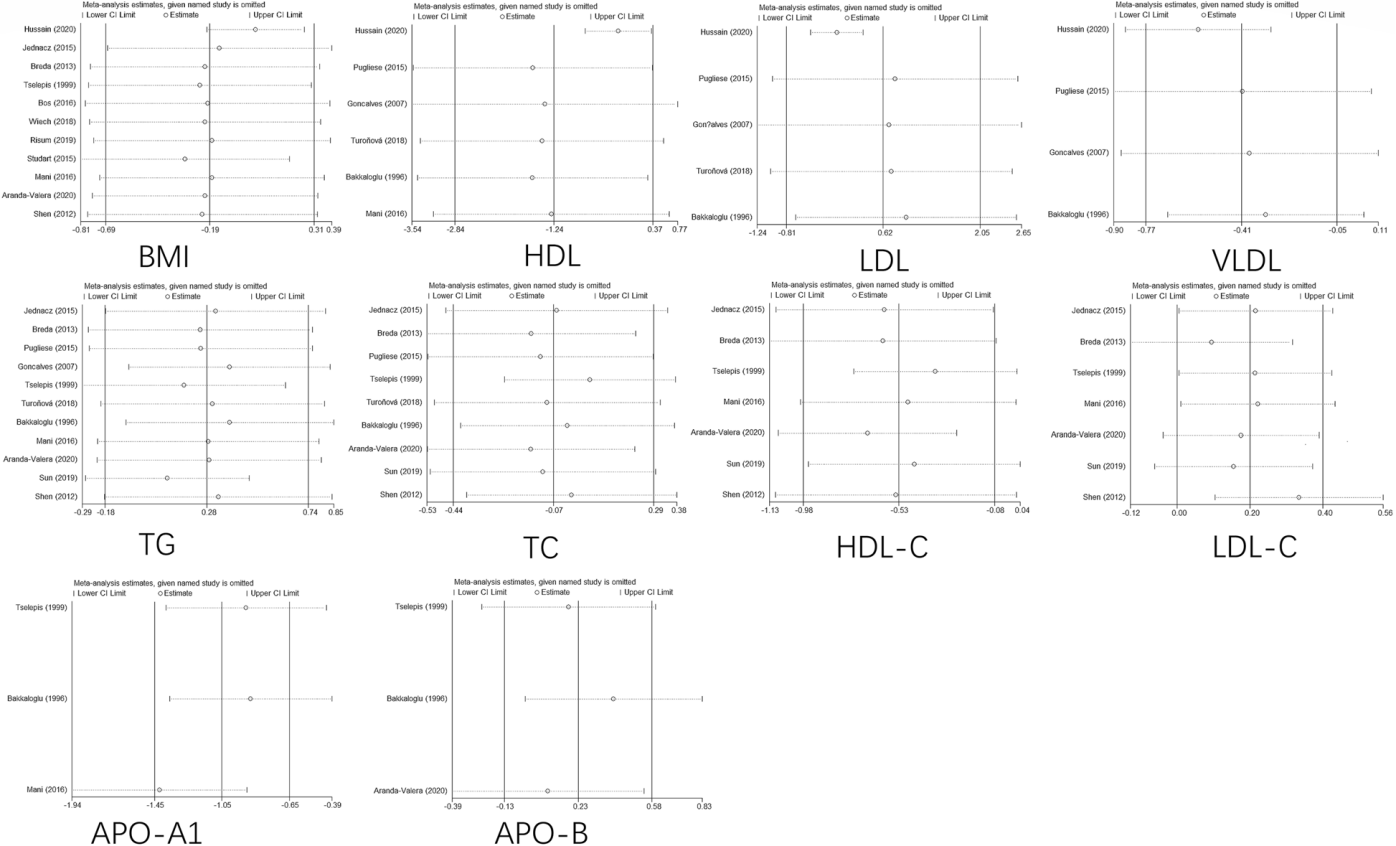
Sensitivity analysis of serum lipids in JIA and healthy controls

**Fig. 13:**
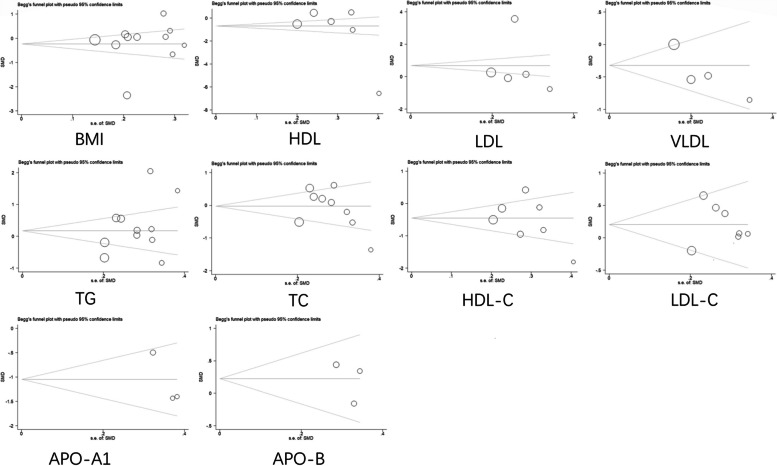
Begg’s funnel plot with pseudo 95% confidence limits

**Fig. 14:**
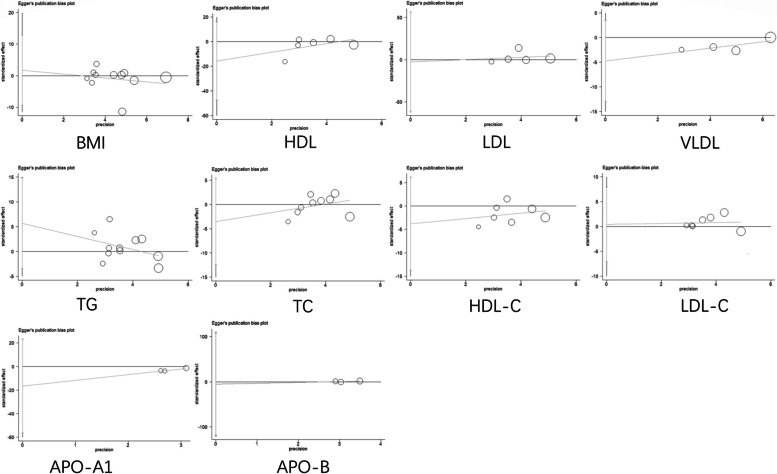
Egger’s publication bias plot
